# Platelet Activating Factor-Induced Ceramide Micro-Domains Drive Endothelial NOS Activation and Contribute to Barrier Dysfunction

**DOI:** 10.1371/journal.pone.0075846

**Published:** 2013-09-27

**Authors:** Sanda Predescu, Ivana Knezevic, Cristina Bardita, Radu Florin Neamu, Viktor Brovcovych, Dan Predescu

**Affiliations:** 1 Department of Pharmacology and Medicine, Division of Pulmonary and Critical Care, Rush University, Chicago, Illinois, United States of America; 2 Division of Pulmonary, Allergy, and Critical Care Medicine, Emory University School of Medicine, Atlanta, Georgia, United States of America; 3 Department of Pharmacology, University of Illinois, Chicago, Illinois, United States of America; University of Illinois College of Medicine, United States of America

## Abstract

The spatial and functional relationship between platelet activating factor-receptor (PAF-R) and nitric oxide synthase (eNOS) in the lateral plane of the endothelial plasma membrane is poorly characterized. In this study, we used intact mouse pulmonary endothelial cells (ECs) as well as endothelial plasma membrane patches and subcellular fractions to define a new microdomain of plasmalemma proper where the two proteins colocalize and to demonstrate how PAF-mediated nitric oxide (NO) production fine-tunes ECs function as gatekeepers of vascular permeability. Using fluorescence microscopy and immunogold labeling electron microscopy (EM) on membrane patches we demonstrate that PAF-R is organized as clusters and colocalizes with a subcellular pool of eNOS, outside recognizable vesicular profiles. Moreover, PAF-induced acid sphingomyelinase activation generates a ceramide-based microdomain on the external leaflet of plasma membrane, inside of which a signalosome containing eNOS shapes PAF-stimulated NO production. Real-time measurements of NO after PAF-R ligation indicated a rapid (5 to 15 min) increase in NO production followed by a > 45 min period of reduction to basal levels. Moreover, at the level of this new microdomain, PAF induces a dynamic phosphorylation/dephosphorylation of Ser, Thr and Tyr residues of eNOS that correlates with NO production. Altogether, our findings establish the existence of a functional partnership PAF-R/eNOS on EC plasma membrane, at the level of PAF-induced ceramide plasma membrane microdomains, outside recognized vesicular profiles.

## Introduction

Platelet activating factor (PAF) is one of the most potent phospholipids released by a diversity of cell types [1–4] upon activation by a variety of agonists [4–6]; its release results in a marked increase in vascular permeability at the level of different vascular beds [7–10]. PAF effects on endothelial cells (ECs) are mediated through the PAF-receptor (PAF-R), a G-protein coupled receptor localized on the plasma membrane, in much larger amounts inside the cell [9] and on the nuclear membrane [11]. When stimulated, endothelial PAF-R activates many enzymatic systems [12–14] leading to diverse cellular responses such as cytoskeletal rearrangements [10,12], enlargement of caveolae, generation of fenestrations and extensive filopodia [9,15], mobilization of Ca^2+^ [16], generation of inositol 1,4,5-triphosphate [17] and production of nitric oxide (NO) [18].

The EC-generated NO, known to control many vital vascular functions [19–22] is produced by the activation (phosphorylation/dephosphorylation) of endothelial nitric oxide synthase (eNOS). Evidence indicates that eNOS activity is complex and tight regulated; its expression, biochemical activity and subcellular localization controls NO production and thus, NO functional efficacy [23–25]. The membrane association of eNOS is required for its phosphorylation and activation by different stimuli [26–28], including PAF [29]. However, PAF-induced NO production after eNOS activation should involve PAF-R direct interaction with the enzyme; still, this interaction was not yet demonstrated. Available data on the spatial relationship between the two proteins point to their residence in separate domains of endothelial plasma membrane. PAF-R resides all over plasma membrane outside of any identifiable vesicular carriers, while eNOS is rationalized to be associated with the plasma membrane of caveolae [30].

While PAF-induced NO was shown to participate in alteration of vascular permeability [18,29,31], PAF-triggered mechanism(s) capable of generating at the cellular level enough force to open the endothelial barrier, just started to be described. Recent work [10] has established that PAF-induced increase vascular permeability uses changes in endothelial actin polymerization/depolymerization, independent of the classic mechanism of myosin light chain (MLC) phosphorylation. This PAF-driven shift in actin status, able to provoke the movement of minute regions of the cell, the interendothelial junctions included, as a molecular process is a much faster and a more effective than the classical mechanism of cellular contraction mediated by the MLC kinase. The early actin polymerization (more F-actin) is a short lived process (~10 min) accounting only for the initial opening of interendothelial junctions, and thus the subsequent actin depolymerization (more G-actin) triggered by unknown mechanisms, is the factor which swells and maintains the opening of endothelial barrier. As actin depolymerization could be the consequence of NO production [32] we decided to study the functional and spatial relationship between PAF-R engagement and NO production by mouse ECs.

In this study we: i) provide data documenting the existence of a spatio-temporal relationship between PAF-R and eNOS on the lateral plane of plasma membrane, ii) add a quantitative estimate of their subcellular distribution, iii) demonstrate that eNOS is activated as a consequence of PAF-R engagement in a ceramide plasma membrane micro-domain, and iv) show that PAF-R triggered NO production is a consequence of eNOS activation by several cellular enzymes, taking place into a newly formed plasma membrane ceramide micro-domain.

## Materials and Methods

### Materials

The reagents were obtained as follows: PAF, PAF-R antagonist (BN 5202, also known as Ginkolide B), L-NAME (N^G^-Nitro-L-arginine-methyl ester-HCl), and PP2 inhibitor from Enzo Life Sciences, Inc. (Farmingdale, NY); H_2_O_2_, glycerol, benzamidine, phenylmethylsulphonyl fluoride (PMSF), sodium pyrophosphate, bovine serum albumin (BSA), desipramine hydrochloride, protease inhibitors cocktail, poly-lysine solution (tissue culture grade), and Hank’s solution from Sigma-Aldrich (St. Louis, MO), while the mouse albumin (MSA), protein A/G immunobeads, wortmanin, LY294002, Ro-32-0432 and 4,5-Diaminofluorescein Diacetate (DAF 2DA) from EMD-Millipore (Rockland, MA); Triton X-100, Nonidet P-40 (NP-40), dodecyl-sulphate sodium salt (SDS), Tween 20, nitrocellulose membranes (NC), and all chemicals for electrophoresis from GE Healthcare (Pittsburgh, PA); the kits for protein determination (BCA Protein Assay - Reducing Agent Compatible, micro-plate format) and for enhanced chemiluminescence (ECL) from Thermo, Fisher Scientific (Rockford, IL); the 105 MultiSort Kit (PE) for isolation of mouse ECs form Miltenyi Biotech Inc (Auburn, CA), while for NO detection the Nitric Oxide Quantitation Kit from Active Motif (Carlsbad, CA). The following primary antibody (Ab) were used: anti-actin from Sigma-Aldrich; anti-cSrc, anti-phospho (p)-cSrc, anti-p-Thr-495, and anti-p-Ser-1177 eNOS from EMD-Millipore, anti-human PAF-R from Cayman Chemical (Ann Arbor, MI); anti-eNOS (polyclonal and monoclonal) from BD Biosciences (San Jose, CA); anti-Ti1 (H-180) and anti Tie 2 (H-176) from Santa Cruz Biotechnology (Santa Cruz, CA); Anti-p-Tyr (clone PY20), the secondary Abs (Alexa 488, Alexa 559, and Horse Radish Peroxidase conjugated), acetylated Low Density Lipoprotein, labeled with 1,1'-dioctadecyl - 3,3,3\',3\'-tetramethyl-indocarbocyanine perchlorate (DiL-Ac-LDL) from Invitrogen (Grand Island, NY). Affinity purified F(ab’)2 goat anti-mouse and donkey anti-rabbit IgGs, used as reporter Abs for EM immunocytochemistry, were from Bethyl Laboratories Inc. (Montgomery, TX), while ceramide Ab (clone MID 15B4) from Enzo Life Sciences, Inc (Farmingdale, NY). Collagenase type 1 sterile from Worthington Biochemical (Lakewood, NJ); Dr. Donna Stolz, (Pittsburg University), kindly provided the suspension of colloidal silica.

### Ethics Statement of Animals Usage

All experimental procedures involving Black C57 male mice (3-5 weeks old; Jackson Laboratories), acclimatized at Rush animal facility, used under direct supervision of Rush Institutional Animal Care and Use Committee were performed under anesthesia and all efforts were made to minimize suffering. All mouse studies adhered to APS’s Guiding Principles in the Care and Use of Vertebrate Animals in Research and Training and were performed according with the protocol 11-009 approved by the IACUC from Rush University. Preliminary experiments using 10 male and 10 female mice, age matched indicated no statistical differences in the responses related to the gender; therefore male mice were used throughout this study.

### Isolation and characterization of ECs from murine pulmonary vascular bed

For mouse pulmonary artery ECs (PAECs) isolation, a number of 20 mouse pulmonary artery segments were dissected, cleaned of surrounding connective tissue, and chopped into small (4 x 4 mm) pieces that were washed in complete media containing 2x concentration of antibiotics (Pen-Strep + Antibiotic antimycotic) and finally oriented with the endothelium toward the bottom of 35 mm Petri dish pretreated with a mixture of collagen/fibronectin/MSA. ECs media was added just to cover the pieces that were untouched for 120h. Afterwards, the tissue pieces were switched to normal ECs media. When the growing of cells with endothelial morphology was vigorous enough, they were cloned using sterile glass inserts and trypsin. Cloned cells, when confluent were dislocated with EDTA, and then panned on Petri dishes coated with Tie1/Tie2 Abs, the non-adherent cells removed, while the adherent ones were released by incubation in acidified media (M199/EGM2, pH 3.5, 20 min). Cells were recovered by centrifugation (10 min, 4°C, 1500g), and plated onto collagen/fibronectin/MSA coated plates.

Mouse microvascular ECs (MVECs) were isolated from 10-16 pairs of murine lungs washed blood free, hand-minced and dissociated by incubation with 1% collagenase type II (45min, 37°C) under constant stirring. The primary cell suspension was panned using Petri dishes coated with an anti-Tie1/Tie2 Abs mixture (4h, RT); the non-adherent cells were removed and the adherent ones were released with trypsin. Further the isolation of MVECs was done using CD105 magnetic beads as per manufacturer instructions. The isolated mouse ECs were routinely checked by fluorescence microscopy for the presence of Factor VIII, DiL-Ac-LDL uptake and their endothelial phenotype was confirmed by EM. Contaminating lymphatic endothelia and fibroblasts were evaluated by immunostaining for podoplanin and fibroblast specific protein, respectively. Established mouse PAECs and MVECs lines were used between passage 5-7 and grown routinely in complete endothelial media (M199/EGM2 1:1 containing 10% FCS + 10% mouse serum, 5mM glutamine, 20mM HEPES, 20µg/mL ciprofloxacin, 2mM hydrocortisone, 5ng/mL VEGF, 4ng/mL insulin/transferin/sodium selenite, 50ng/mL heparin, 30 µg/mL ascorbic acid and 1% mouse brain extract prepared by us, as in [33].

The functionality of isolated mouse *ECs* was checked by measuring trans monolayers electrical resistance (TER) under non-stimulated conditions and after PAF challenge using an electric resistance cell-substrate impedance sensor (ECIS, Applied BioPhys). Briefly, confluent monolayers of mouse MVECs and/or PAECs were seeded onto ECIS 8W10E or 8W10E culture plastic-ware pre-coated with 0.1% gelatin and TER was measured as described [34]. The interaction of MSA with the mouse ECs was determined by seeding cells on Costar 0.4 µm Transwell filters and when confluent, cells were exposed to dinitro-phenylated-MSA (MSA-DNP; 0.5 mg/mL) for quantitative assessment of monolayer permeability and/or gold-tagged-MSA (Au-MSA; 1 mg/mL) for morphological surveys.

For quantitative assessment of monolayer permeability the mouse ECs were incubated with MSA-DNP and then triplicate samples from the lower chamber at different times points were used to determine the amounts of MSA-DNP as in [35,36]; its clearance was calculated as in [37], and the values are expressed as ng MSA-DNP/0.1mL/1h after normalization to an empty filter.

### Cell treatment, cell and tissue lysates

Treatment of cultured mouse ECs with the enzyme inhibitors was performed on cells starved of growth factors (EGM-2 + 0.5% FCS for 8h). Cells and blood-free mouse lung lysates were prepared using 50 mM Tris-HCl pH 7.4, 1 mM EGTA, 1% SDS, protease inhibitors for 1h, at RT, under continuous agitation, and the ensuing lysates were clarified by centrifugation (30 min, 40,000 rpm, 4°C) in a Beckman Optima Max-XP ultracentrifuge). Phosphorylated proteins were solubilized in 50mM Tris-HCl pH 7.5, 1 mM EDTA, 1 mM EGTA, 2 mM sodium orthovanadate, 50 mM sodium fluoride, 1% Triton X100, 1% SDS and protease inhibitors.

### Tracers and EC monolayers preparation for transmission electron microscopy (TEM)

Colloidal Au particles with an average diameter of 6-8nm, 15nm and 22nm Au particles were obtained as in [38]. Au tagged-MSA (Au-MSA) was obtained by stabilizing colloidal Au suspensions (8nm) with MSA (800 µg/ml) while the ceramide Au conjugates were prepared by adsorbing the affinity-purified immunoglobulin (50 µg/ml) on 22 nm colloidal Au particles, and the secondary tagged F(Ab’)2 by adsorbing on 6 or 15 nm gold particles. For Au-MSA as well as for all Ab-Au tracers, a secondary stabilization of Au suspensions was obtained by 200 µg/ml poly l-glutamic acid, the stabilized solutions were kept as stock until used and diluted just before use in PBS to *A*_520_ = 0.2–0.3 for Ab-Au and an *A*_520_ = 1 for A-Au. MSA was tagged with DNP as in [35]. Quantitative assessment of MSA-DNP transport was done by ELISA as in [36], using a standard curve generated with known amounts of the same tracer. For TEM evaluation of mouse ECs, the monolayers were fixed in 2% paraformaldehyde (PFA) + 0.5% glutaraldehyde (GA) + 1% tannic acid in 0.1 PIPES, pH 7.2) for 30 min at RT; all specimens were post-fixed in 2% OsO_4_ in acetate veronal buffer, pH 6.8, for 30 min on ice as in [42]. Fixed ECs were stained for 30 min with 7.5% uranyl-magnesium acetate, dehydrated through increasing concentrations of ethanol, then infiltrated with a mixture of 100% alcohol: Epon (1:1) for 1h and embedded in Epon 812. Embedded specimens were cured for 72 h at 90°C, and sections, ~60 nm thick, stained with 7.5% UA for 5 min and saturated lead citrate for 3 min were examined and photographed in a Jeol-1220 electron microscope.

### Morphometric Analysis

Epon blocks (6-8) were employed for thin sectioning, and six grids per block, 15–25 sections/grid were examined. Sections (60–70 nm) were obtained at random, and only cells with a full profile on the grid mesh were photographed. The images were acquired with a Gatan charge-coupled device camera. A total of 72 images for every condition, at the final magnification of x28, 000, were stacked as a queue and grid no. 3 from the Stereology Toolbox program (Morphometrix) was used to quantify the main endothelial features.

### Immunochemical methods

*For immunoblotting*, 20–80 µg protein per lane were loaded on a 5-20% SDS PAGE minigel, run at 150V, and transferred to NC membranes followed by incubation with the primary and secondary Abs and processed as in [39]. The reaction was visualized by ECL and HyBlot CL films. When the phosphorylation of different proteins was monitored, the NC membranes were blocked in 1% non-fat dry milk, 1% BSA, 0.05% Tween 20, 2 mM sodium orthovanadate, 50 mM sodium fluoride, and 2 mM sodium pyrophosphate, then incubated with the phospho-specific Abs followed by appropriate reporters, washed, and developed as above. When needed, the NC membranes were striped for 30 min at 50°C (1M Tris-HCl pH 6.8, 10% SDS, 10 mM β-mercaptoethanol), and reprobed with Abs against the non-phosphorylated proteins. *For immunoprecipitation*, cell lysates (1 mg/ml total protein) were precleared with 50 µl of Protein A/G slurry, centrifuged and then the supernates were incubated with the primary Abs. Antigen-Ab complexes were recovered by incubation with 20 µl of Protein A/G slurry and centrifugation, then solubilized (2xSDS sample buffer), and resolved by SDS-PAGE. *For immunofluorescence*, confluent ECs monolayers were washed with ice-cold PBS, fixed/permeabilized with methanol (5min, -20°C), blocked with 1% BSA in PBS (PBS-BSA) and incubated with the primary Abs (1h, RT), washed with 0.1% BSA in PBS, incubated with the corresponding secondary Abs diluted in PBS-BSA (1h, RT), washed again, mounted with Prolong-antifade medium, and examined with an Zeiss Axio-Imager M1 microscope.

### Fluorescence microscopy and TEM on plasma membrane cortices

Plasma membrane patches were prepared, labeled and used as in [40]. Briefly, for fluorescence microscopy, confluent monolayers of ECs grown on poly-lysine-coated glass cover slips were shortly washed in sonication buffer (20 mM HEPES pH 7.2, 120 mM potassium glutamate, 20 mM potassium acetate and 2mM EGTA) and then subjected to brief sonication. Plasma membrane patches attached to the coverslips were fixed in 4% PFA in PBS, quenched in 50 mM NH_4_Cl in PBS, and then sequentially incubated for 30 min with the primary Abs, followed by 45 min incubation with the reporter Abs. For EM immunogold labeling, plasma membrane patches were prepared by brief sonication of ECs grown on poly-lysine coated gold EM grids. Plasma membrane sheets still attached to the grids after sonication were fixed for 15 min in 2% PFA, quenched with 1% BSA in TBS, then incubated with the primary Abs (diluted 1:100 in 1% MSA in TBS), followed by 15 nm Au-conjugated first reporter Ab (diluted in 0.1% MSA in TBS) and for double immunostaining the cortices were reacted with 5 nm Au-conjugated second reporter Ab diluted as above. Control experiments were performed to establish the specificity of staining by omitting the first Abs, while for defining the background, we used isotype matched Abs. The grids were washed by transfer through successive drops of TBS, then post-fixed in 2% GA (15 min, RT), and stained with uranyl acetate and lead citrate. The EM grids were examined in a Jeol 1220 TEM equipped with a Gatan CD camera. To quantify the distribution of Au particles, we used three sets of micrographs (72 per set representing ~ 372 µm^2^ of plasma membrane surface). The distribution of Au clusters, in double immunostaining experiments, was estimated using at least 175 clusters per condition, while the quantitative analysis of colocalization on plasma membrane patches by fluorescence microscopy was performed as described in [41], and for TEM studies as in [42]. When the distance between different sizes Au particles was < 20 nm we consider the generating molecules as being colocalized (residing in the same space).

### Preparation of subcellular fractions by silica coating

The subcellular fractionation of confluent monolayers of mouse ECs using the silica methodology was done as in [43]. Briefly, monolayers of ECs were washed with coating buffer (135 mM NaCl, 20 mM HEPES, 1 mM Mg^2+^ and 0.5 mM Ca^2+^), then coated with 1% (w/v) cationic colloidal silica in coating buffer. The monolayers were treated with 1mg/mL polyacrylic solution in coating buffer, pH 5.0, and then with domain lysis buffer (2.5 imidazole pH 7.5, 1 mM Mg^2+^, 0.5 mM Ca^2+^, protease inhibitor cocktail, 5µg/mL E64 + 1,10-phenantroline). The monolayers covered with silica were unroofed, by squirting the cell monolayer at ~ 45° angle using a 5 mL syringe fitted with a 20G needle. The domain lysis buffer-lysate solution was saved and used for isolation of apical plasma membrane, internal membranes, nuclear fraction as well as soluble fraction. The membranes contained in the internal membranes were pelleted by ultracentrifugation (100,000g for 60 min at 4°C), the pellet was washed twice by centrifugation in the same conditions and used for biochemical analyses, while the supernate, representing the soluble fraction was collected separately. The basolateral membrane domain which remains attached to the dish and all other subcellular fractions were solubilized with 2% SDS in lysis buffer.

### Visualization and quantitation of NO production

The amounts of NO were quantified using the kit from Active Motif according with manufacturer terms. Real-time production of NO was determined by measurement of NO with a porphyrinic microsensor consisting of 5–7 carbon fibers (diameter 0.2µm), as in [44]. The sensor, part of a three-electrode system (the porphyrinic NO-sensitive electrode, counter platinum electrode, and calomel reference electrode), was positioned with the help of a micromanipulator close to the ECs culture surface (20 ± 1 µm) and used at a constant potential of 700 mV. For recordings, the cultures were changed to phenol red-free and serum-free DME/F-12 medium, and then PAF-induced NO generation was recorded using a computer-based Camry VP600 potentiostat. In situ visualization of NO production within the cells was performed as in [45] using DAF-2 DA, in order to efficiently load the cells. To provide direct evidence of NO production by both types of mouse ECs, experiments were performed in the presence of L-NAME inhibitor, which was kept throughout the procedures used for NO imaging and recording.

### Determination and visualization of ECs surface ceramide

Monolayers of ECs were fixed (1% PFA in TBS), then incubated with 2µg/mL anti-ceramide Ab (45-60 min, RT) and washed 3x with TBS as in [46]. Cell-bound Ab was released by 30 sec treatment with ice-cold 100 mM glycine, pH3.5, neutralized with 1M NaOH, and cell lysates spotted onto NC membrane using a dot-blot apparatus followed by incubation with anti-goat IgM HRP-conjugated. The immunoreactive spots were detected by ECL and quantified using an Alpha Imager HP-MultiImage II system. Concomitant detection of PAF-R and ceramide was done, using the protocol from [35,36] modified by us for surface double immunolabeling. Mouse ECs grown in 35-mm plastic Petri dishes were washed (PBS; three times for 2 min), fixed (PBS containing 2% PFA and 0.2% GA; 30 min, RT), washed again (PBS + 0.2% ovalbumin), 3 x 2 min), and then reacted with PAF-R Ab coupled to 22nm Au particles, diluted in the same buffer, for 12 h, at 4°C. After washing (PBS + 0.2% ovalbumin, 5 x 5 min), the cells were reacted with ceramide Ab (same Ab used for ceramide detection on NC Membranes) coupled to 6 nm Au particles, for 4 h, at 8°C. The cells were again washed, fixed with 0.2% GA in PBS, and subjected to the standard Epon embedding as described above. The specificity of the Ab labeling was demonstrated using a nonspecific, isotype-matching Ab or by omitting PAF-R or ceramide Abs. Thin sections cut from blocks mounted on nickel grids were examined and micro-graphed in the Jeol 1220 TEM.

### Statistical analysis

Data obtained by Western blotting and the normalization of ECIS experiments were compared using one-way analysis of variance and Student’s *t* test with a Bonferoni correction for multiple comparisons. All data are expressed as mean ± standard mean error. For MSA permeability, data were analyzed by one-way ANOVA followed by a post hoc Dunnett’s test. *P* values < 0.05 were considered to have statistical significance.

## Results

### PAF exposure increases the transport of macromolecules across murine cultured ECs monolayers

**Figure 1:**
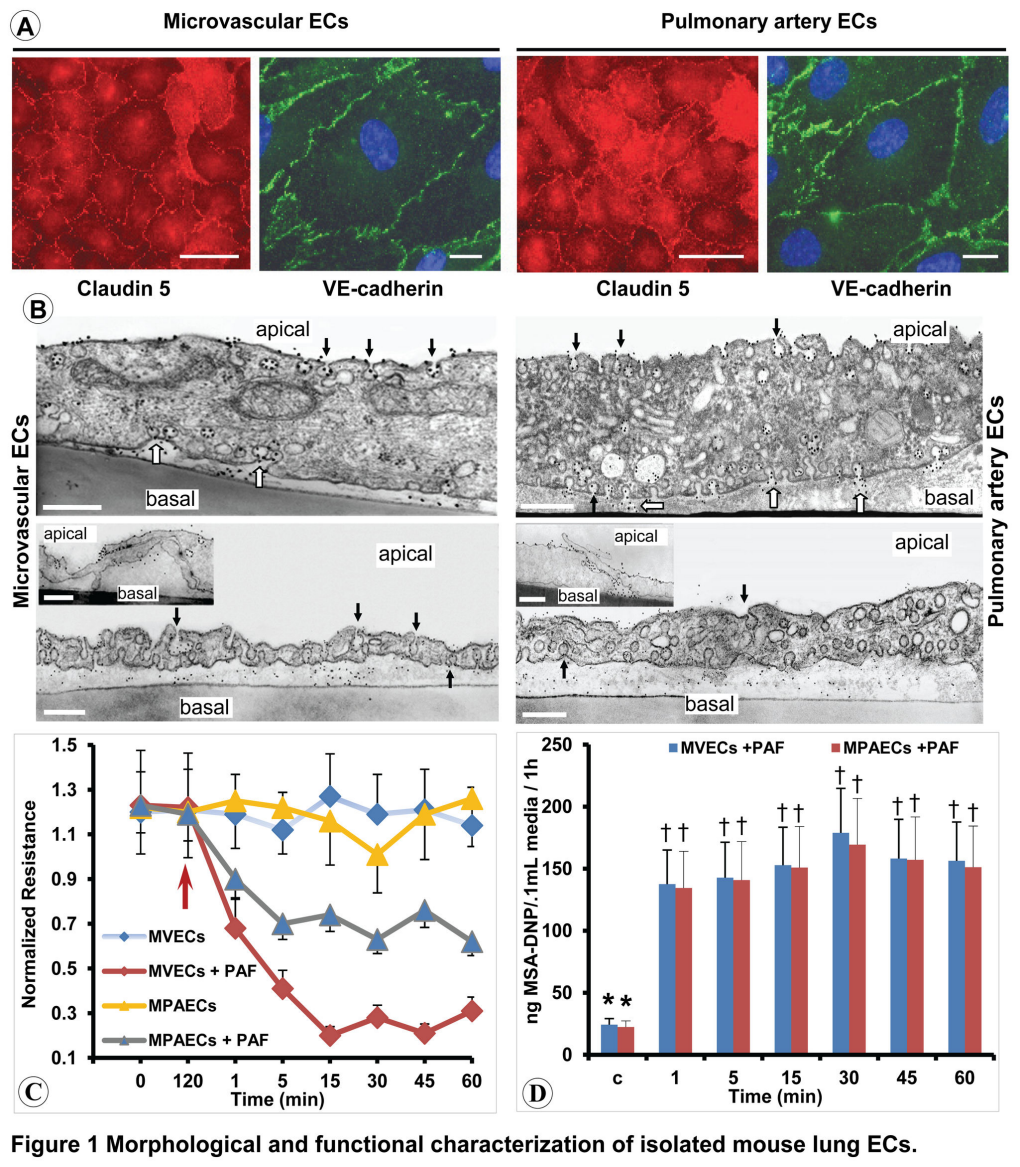
Morphological and functional characterization of isolated mouse lung ECs. (**A**) Representative micrographs of MVECs and PAECs labeled by claudin-5 and VE-cadherin Abs, followed by Fluorofluor-conjugated appropriate reporters. Bars: 20 µm; n= 9. (**B**) ECs show numerous caveolae (arrows), clathrin-coated vesicles (CCV), mitochondria (M), Golgi apparatus (G), endosomes (E), as well as interendothelial junctions (insets). They actively transport MSA coupled either with Au (upper panels) or DNP (lower panels). Au particles label caveolae on the apical side of ECs (arrows) caveolae in transit across ECs as well as caveolae open to the basal front (open arrows). Bars = 300 nm; n = 6 for every cell type and tracer. (**C**) ECs were exposed to PAF (120 min; arrow) and TER was monitored over time. Data are expressed as means ± SE and are representative of 6 independent experiments per condition. (**D**) The amounts of MSA-DNP tracer transported in the lower chamber as detected by ELISA in 5 independent experiments. Values are means ± SE. * P < 0.05 when ECs treated with PAF were compared to control, as determined by 1-way ANOVA followed by Dunnett’s test. There is no statistical difference between two cell types in regard of trans-monolayer permeability^#^.

To demonstrate the effects of PAF on NO production by ECs, we first isolated mouse lung MVECs and PAECs and characterized them morphologically by fluorescence microscopy and TEM and functionally by measurements of TER and albumin clearance (Figure 1). Both, murine MVECs and PAECs express claudin-5 and VE-cadherin (Figure 1 A), display numerous caveolae (Figure 1B, arrows), clathrin-coated vesicles, the usual set of intracellular organelles, mitochondria, Golgi apparatus, endosomes, as well as interendothelial junctions (Figure 1B, insets), all morphological characteristics of EC phenotype. For TER measurements, the MVECs and MPAECs grown on gold electrodes were allowed to equilibrate for 2h before addition of PAF; only monolayers with a stabilized baseline were used. Noticeably, because in the initial experiments carried out with isolated PAECs we couldn’t detect modifications in TER after PAF, we have to pretreat this monolayers with indometacin (25µM) in order to obtain biological effects; hence all experiments carried out on isolated PAECs were performed on monolayers treated with indometacin. TER values for mouse PAECs were 14±5 Ω·cm^2^ and for the MVECs 17± 8 Ω·cm^2^, comparable to the TER of HUVECs monolayers used as control which showed an average electrical resistance of 12±5 Ω·cm^2^ (not shown). When murine ECs were exposed to 10^-10^M PAF (Figure 1C, arrow) and TER monitored for 60 min, both cell types responded by decreasing the electrical impedance, Figure 1C, 3 to 4-fold for MVECs and 2-fold for PAECs.

Next, biochemical measurements of albumin clearance by ELISA using DNP Ab and MSA-DNP as tracer indicated that PAF treatment increases 4-6 fold the trans-monolayer transport of the tracer, for both MVECS and PAECs, compared to untreated murine ECs, Figure 1D. When exposed to MSA-DNP, 3 day post-confluence, ECs monolayers allowed the passage of the tracer at rate of 31.6 ± 11.8 ng MSA-DNP detected in 100µL media when normalized to 1h interval. Measurements of MSA-DNP amounts indicated that 1 min after PAF, the lower chamber contains 141.6 ± 9.3 and 136.8 ± 12.1 ng MSA-DNP / 100 µL media for MVECs and PAECs, respectively. By 5 min, we found 147.3 ± 12.4 ng MSA-DNP / 100µL media for MVECs and 139.2 ± 11.7 ng MSA-DNP / 100µL media for PAECs, that reached 151.1 ± 14.5 and 149.3 ± 12.2 ng MSA-DNP / 100 µL media at 15 min. By 30 min the amounts of MSA-DNP in 100 µL media reached 188.9 ± 21.4 ng MSA-DNP / 100 µL media for MVECs and 169.6 ± 16.6 ng MSA-DNP / 100 µL media for PAECs. For the last 30 min we did not detect any increase in the transport of MSA-DNP that remained at 155.2 ± 14.4 ng MSA-DNP / 100 µL (45 min) and 150.8 ± 13.7 ng MSA-DNP / 100 µL (60 min) for MVECs and almost at the same level for PAECs, 153.8 ± 18.8 ng MSA-DNP / 100 µL at 45 min, and 149.8 ± 14.7 ng MSA-DNP / 100 µL at 60 min. Thus, while under basal conditions the monolayers transported on average 32 ng MSA-DNP / 100 µl/1h, when stimulated with 10^-10^M PAF the tracer transport was 4–6 fold increased.

To get more insight into the increased MSA-DNP transport, we assess the cellular transport of macromolecules using either 8nm Au conjugated MSA, [molecular diameter of 18-22 nm] or physiologically relevant molecules (MSA-DNP, molecular diameter of 6nm x 6 nm x 12 nm) detected via post-embedding immunocytochemistry with anti-rabbit IgG conjugated to 5 nm Au particles as in [35,36], followed by EM analyses. We found: i) caveolae open on both sides of the cells (Figure 1B, arrows), as well as the apparently free caveolae, scattered throughout the cytoplasm, labeled by tracer particles, ii) Au particles on the basal side of the cells in phase with caveolar openings (Figure 1B, white arrows), and iii) the basal spaces heavily labeled by MSA-DNP (Figure 1B, lower panels). The interendothelial junctions of both MVECs and PAECs were occasionally able to restrict the passage of MSA-DNP (MVECs, inset), and in the majority of cases (> 85%) they are readily penetrated by tracers as large as Au-MSA (PAECs, inset).

Based on these data we concluded that the isolated murine ECs are endowed with the main structural characteristics of endothelial phenotype and are able to transport molecules presented on their apical side. Even if readily penetrated by tracers as MSA-DNP, the murine vascular ECs have the tendency to form a cellular barrier which usually restricts the passage of Au-MSA. However, when stimulated with 10^-10^M PAF the tracer transport, was 4-6 fold increased on both types of ECs.

### PAF-stimulated NO production

**Figure 2:**
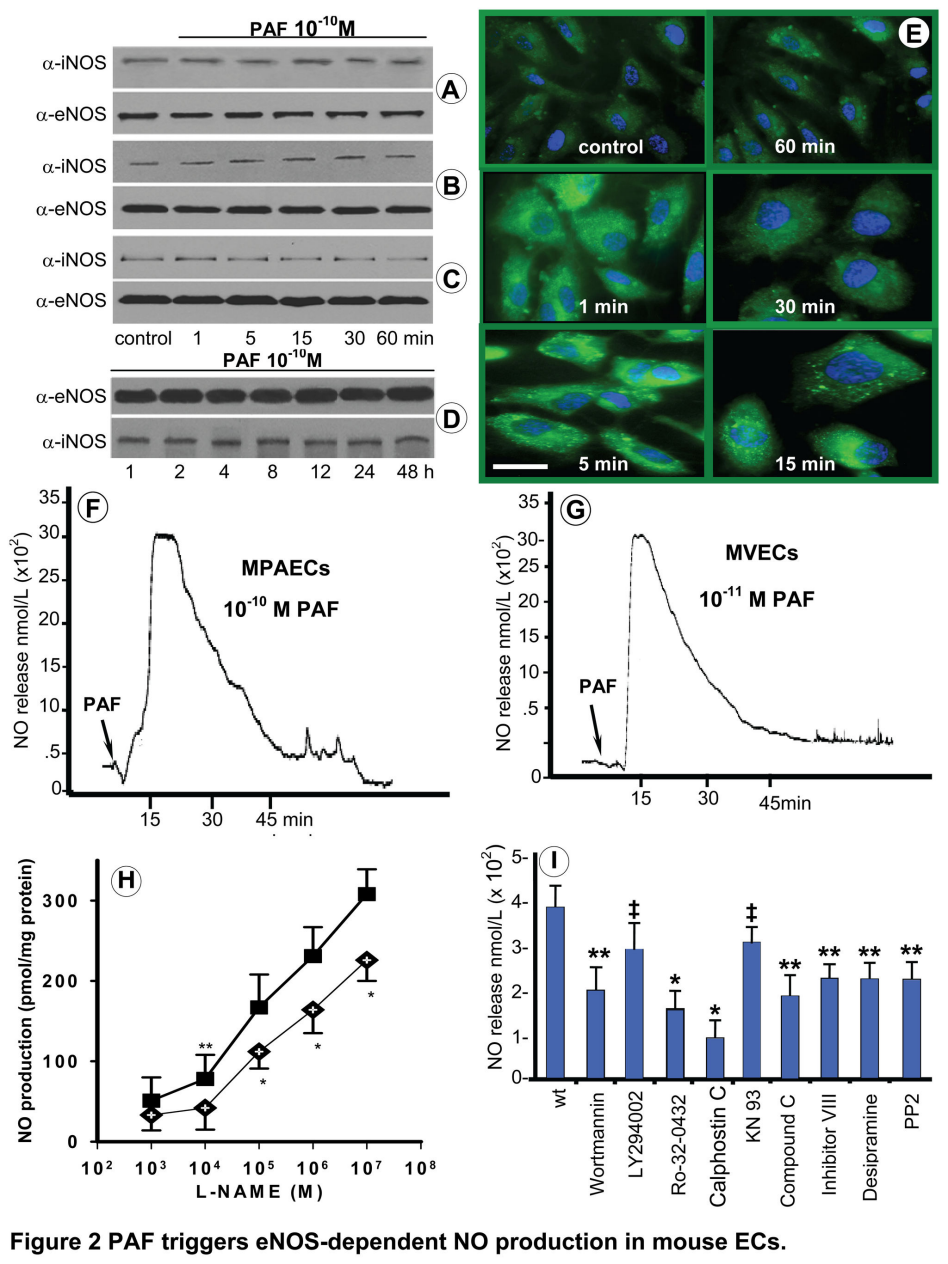
PAF triggers eNOS-dependent NO production in mouse ECs. (**A**) Control and PAF-treated MVECs, PAECs and mouse lung lysates were subjected to SDS PAGE, electrotransfer to NC membranes and Western blot to analyze the expression of iNOS and eNOS enzymes. The results are representative for 6 different experiments. (**B**) In situ NO production by cultured MVECs preloaded with DAF-2 DA. Bar 25 µM, n = 10. (**C, D**) PAF-induced NO production recorded with a porphyrinic sensor on MPAECs and MVECs; n = 12. (**E**) L-NAME inhibition of NO production in, MVECs and PAECs. n = 20 for each type of mouse ECs. * P < 0.02 and **P < 0.05 when determined by 1-way ANOVA followed by post hoc Dunnett’s test for group comparisons. (**F**) The effect of different enzyme inhibitors on NO production recorded in real-time, n = 12 (4 experiments in triplicates). *P < 0.001 compared with wt, ** P < 0.05 when contrasted with wt, and ‡ P > 0.05 when compared with wt.

Because previous studies indicated that PAF has the capacity to induce NO production, we evaluated next whether PAF affects the eNOS and iNOS protein expression in the isolated mouse ECs. Western blot analyses of lysates obtained from cultured MVECs or PAECs (Figure 2A) exposed to 10^-10^ M PAF for up to 60 min using eNOS polyclonal Ab or iNOS monoclonal Ab showed no detectable changes in the protein levels of the two enzymes after 1h treatment. Similar findings were noted for lung lysates of wt-mice exposed to PAF for 60 min or up to 48h (Figure 2A). Then, PAF stimulated NO production was detected either by fluorescence microscopy of DAF-AM loaded ECs monolayers or by using a porphyrinic electrode. PAF treatment of cultured PAECs, pre-loaded with DAF-2DA (10 µM, 60 min, RT), stimulates NO production as soon as 1 min, which peaks between 5-15 min, returns toward basal levels by 30 min and reaches its bottom by 60 min (Figure 2B). Similar results were obtained with DAF-AM loaded MVECs (not shown). However, it should be mentioned that PAF was active on PAECs at 10^-10^M, while for MVECs at 10^-11^M, reality that was holding also in the case of NO production. PAF-induced NO production by PAECs (Figure 2C), or MVECs (Figure 2D), recorded in real time using a porphyrinic electrode, indicated i) the existence of a NO burst into the media, with a maximum at 10-15 min, lasting more than 30 min and returning toward basal levels by 60 min, ii) a similar NO production profile and intensity of the burst for the two pulmonary ECs types, and iii) in both cell types an average NO production of 316 ± 34 nM/L, value that was not statistically influenced by different concentrations of PAF [10^-11^ (305±67 nmol/L), 10^-9^ (327±58 nmol/L), 10^-8^ (342±28 nmol/L), 10^-7^ (361±71 nmol/L) M]. We also found that concentrations of PAF below 10^-11^M did not elicit NO production, while concentrations above 10^-7^ M had cytotoxic effects. The dependence of PAF-induced NO production on eNOS enzyme was established by pretreating mouse ECs monolayers with L-NAME, (Figure 2E), which at a concentration of 10^-4^M was able to blunt NO production by >90% in a concentration-dependent fashion in both types of ECs.

To better define the enzymes utilized by PAF to modulate NO production we used a set of inhibitors known to affect the synthesis outcome of eNOS. Calphostin C and Ro-320432, known inhibitors of PKC were the most potent suppressors of NO production (Figure 2F; 73%, and 56% respectively, compared to the basal production of NO by untreated ECs, considered 100%). Almost in the same range of potency was the Compound C (dorsomorphine) >50%, an inhibitor of AMPK; while the PI3K inhibitors Wortmanin and LY294002 also reduced NO production by <45% and 30% respectively. The inhibitors for AKT – Inhibitor VIII –and for PKA – amide 14-22 – reduced NO production by < 40%, while KN 93, an inhibitor for calmodulin kinase II, had the lowest inhibitory effect (< 10%). Finally, desipramine, an inhibitor of Acid Sphingo Myelinase (ASM), reduced PAF-induced NO production by < 40%, while PP2, an inhibitor of cSrc, diminished NO production by >35%. The specificities, concentration used and the extent of inhibition (%), by different compounds used, are presented in Table 1. For this type of experiments we used MVECs, given the fact that they can be more easily obtained in larger quantities; however, at the beginning, experiments were carried in parallel with both MVECs and PAECs and we find out that the two cell types respond similarly.

**Table 1:** Inhibitors (enzymes and receptor) that affect PAF induced NO production.

**Compound Name**	**Concentration**	**Specificity**	**Inhibition (%)**
**Wortmannin**	1 µM	PI3K, MAPK, MLCK	44.8
**LY294002**	2 µM	PI3K	30
**Ro-32-0432**	200 nM	PKC isoforms	56
**KN 93**	1 µM	CaMK II	8.9
**Calphostin**	75 nM	PKC	73.2
**Compound C**	200 nM	AMPK	52
**Inhibitor-VIII**	500 nM	AKT	39.7
**Amide 14-22**	5 µM	PKA	39.1
**Desipramine**	2 mM	ASM	39.3
**PP2**	50 nM	cSrc	35.7
**BN 52021**	2 µM	PAF-R	Antagonist (>95%)
**L-NAME**	10^-4^ to 10^-7^ M	NOS	98 to 100

All inhibitors (concentrations, specificity) used were chosen based on the technical specifications provided by the supplier. The % inhibition was calculated as detailed in the text; all experiments involving inhibitors were repeated 6 times. Abbreviations used: PI3K - Phosphoinositide 3-kinase; MAPK - *Mitogen*-activated protein kinase; PKC - Protein *kinase* C; CaMK II - Ca^2+^/calmodulin-dependent protein *kinases* II; AMPK - AMP-activated protein kinase; AKT - the kinase encoded by AKT8 gene from the ARK mouse, also known as PKB; PKA - Protein *kinase* A; ASM - acid sphingomyelinase; cSrc - the kinase encoded by the vSrc gene in the chicken Rous sarcoma virus.

### PAF induces a dynamic phosphorylation of eNOS

**Figure 3:**
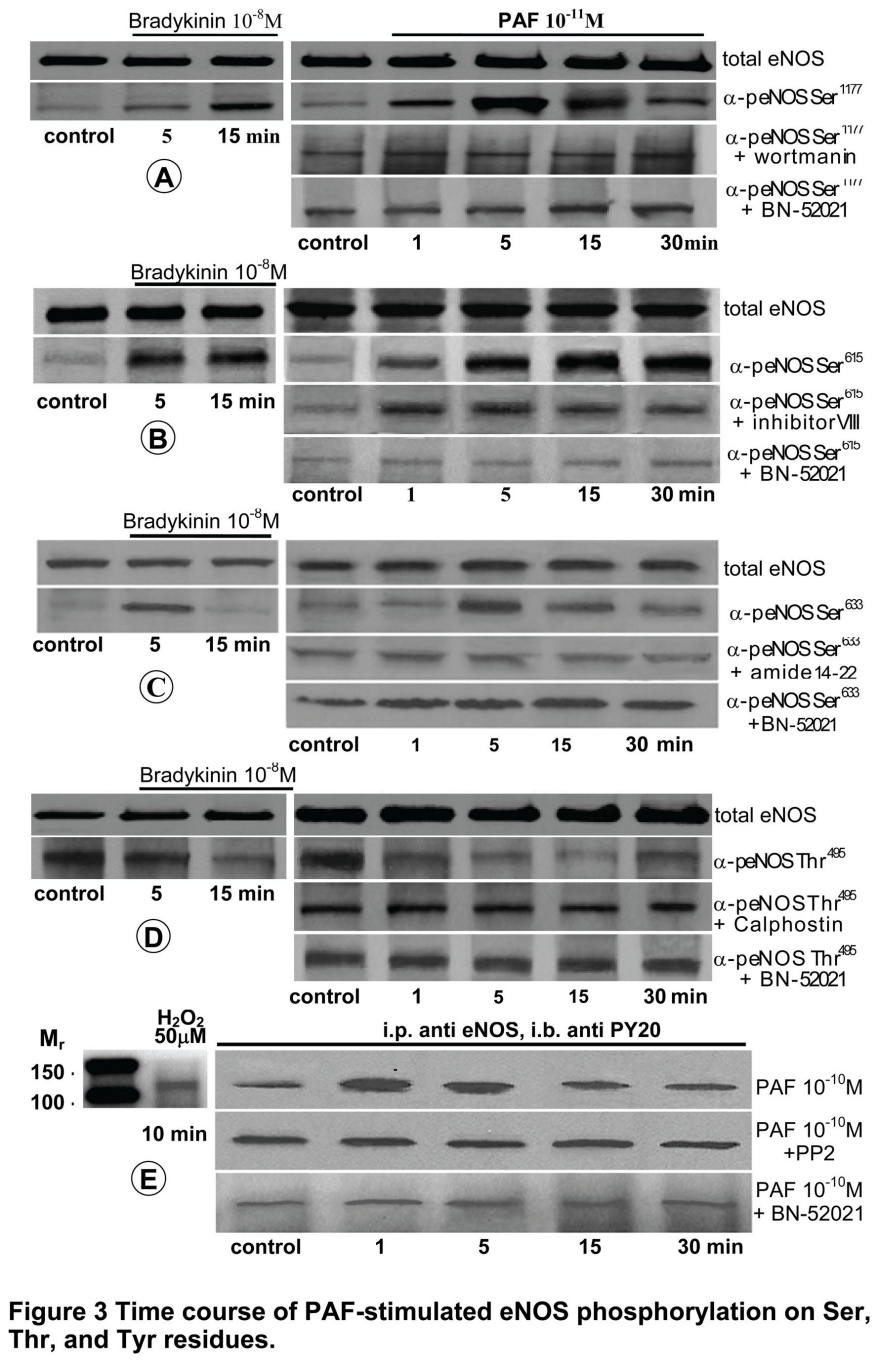
Time course of PAF-stimulated eNOS phosphorylation/dephosphorylation on Ser, Thr and Tyr residues. Representative blots illustrate the phosphorylation status of (**A**) Ser^1177^, (**B**) Ser^615^, (**C**) Ser^633^, and (**D**) Thr^495^ residues of eNOS enzyme in mouse ECs subjected to PAF exposure. The upper panels in A - D show that the total amounts of eNOS do not change. The left panels A - D are positive control showing the effect of bradykinin treatment on total eNOS (upper panel) and on the phosphorylation of Ser^1177^ (**A**), Ser^615^ (**B**), Ser^633^ (**C**) and Thr^495^ (**D**) (lover panel) in the isolated ECs. (**E**) Phosphorylation status of eNOS Tyr as determined by immunoprecipitation (IP) with eNOS Ab followed by Western blot (WB), with phospho-Tyr Ab PY-20 (upper panel). The panel on the left illustrates that H_2_O_2_-induced phosphorylation of eNOS Tyr also occurs in mouse ECs. All blots are representative for 4-8 experiments for each mouse EC line.

As eNOS can be phosphorylated on Ser, Thr and Tyr residues, the effects of PAF on eNOS phosphorylation were assessed using either phospho-site specific Abs, when available, or immunoprecipitation coupled with immunoblotting for Tyr phosphorylation for which there is no site specific phospho Abs. Thus, total cell lysates obtained from control and mouse ECs monolayers treated with 10^-11^ PAF along with monolayers pretreated with kinase inhibitors or BN 52021(a powerful PAF-R inhibitor) were resolved by SDS-PAGE and then subjected to Western blot analyses. PAF challenge induced a dynamic phosphorylation / dephosphorylation of eNOS (Figure 3) dependent on receptor engagement and kinase activity. PAF induced a rapid and sustained phosphorylation of Ser^1177^; it starts at 1min, peaks at 5 min and returns toward basal levels by 30 min. Ser^1177^ phosphorylation is inhibited by 60 min pretreatment with wortmanin (1 µM), not a very specific kinase inhibitor (Figure 3A, Table 1). Ser^615^ phosphorylation is quick, starts after 1 min, but lasts beyond 30 min; its activation is inhibited by Inhibitor VIII (5µM, 30 min; Figure 3B) and/or Compound C (20 µM, 30 min; Table 1), suggestive of AKT and/or AMPK involvement. Phosphorylation of Ser^633^ starts at 5 min, is transitory, lasts less than 30 min and is inhibited by myristoylated form of amide 14-22 (5µM, 30 min) Figure 3C, indicative of PKA involvement in controlling the activity at this site (Table 1). PAF triggered a dephosphorylation of Thr^495^ (Figure 3D), effectively (>70%) abolished by 45 min pretreatment with Calphostin (75nM), but not to the same extent (~56%) by the more specific Ro-32-0432 (200nM) PKC inhibitor (Table 1), suggesting the involvement of different PKC isoforms in the activation status of this site. Our results also show that PAF induced a transient phosphorylation of eNOS Tyr residues, as detected by immunoprecipitation with eNOS Ab followed by immunostaining with a PY-20 Ab (Figure 3E). Similar results were obtained when the immunoprecipitation was performed with anti PY-20 Ab followed by immunoblotting with anti eNOS Ab (not shown). Phosphorylation of eNOS Tyr residues was partially (~40%) prevented by pretreating the monolayers with PP2 (50 nm, 45 min), as shown in Table 1, suggesting that more than c-Src kinase family are involved in determining eNOS Tyr phosphorylation status. H_2_O_2_-induced phosphorylation of eNOS Tyr in mouse ECs was used as positive control (Figure 3E, left panel). Moreover, BN 52021 (2 µM, 30 min), a specific PAF-R inhibitor [47] blocked phosphorylation/dephosphorylation of all sites (Figure 3 A to E). We also show that the isolated mouse ECs respond to bradykinin (10^-8^M), a known mediator of eNOS phosphorylation, (A -D, lower left panels), which does not change the total amounts of eNOS (upper left panels). Thus, the dynamic aspect of phosphorylation events corroborated with the effect of different enzyme inhibitors on NO production led to the conclusion that PAF-induced NO production by ECs is directly dependent on eNOS post-translational alteration induced by known kinases activated by PAF-R engagement.

### PAF-induced ceramide production in murine ECs

**Figure 4:**
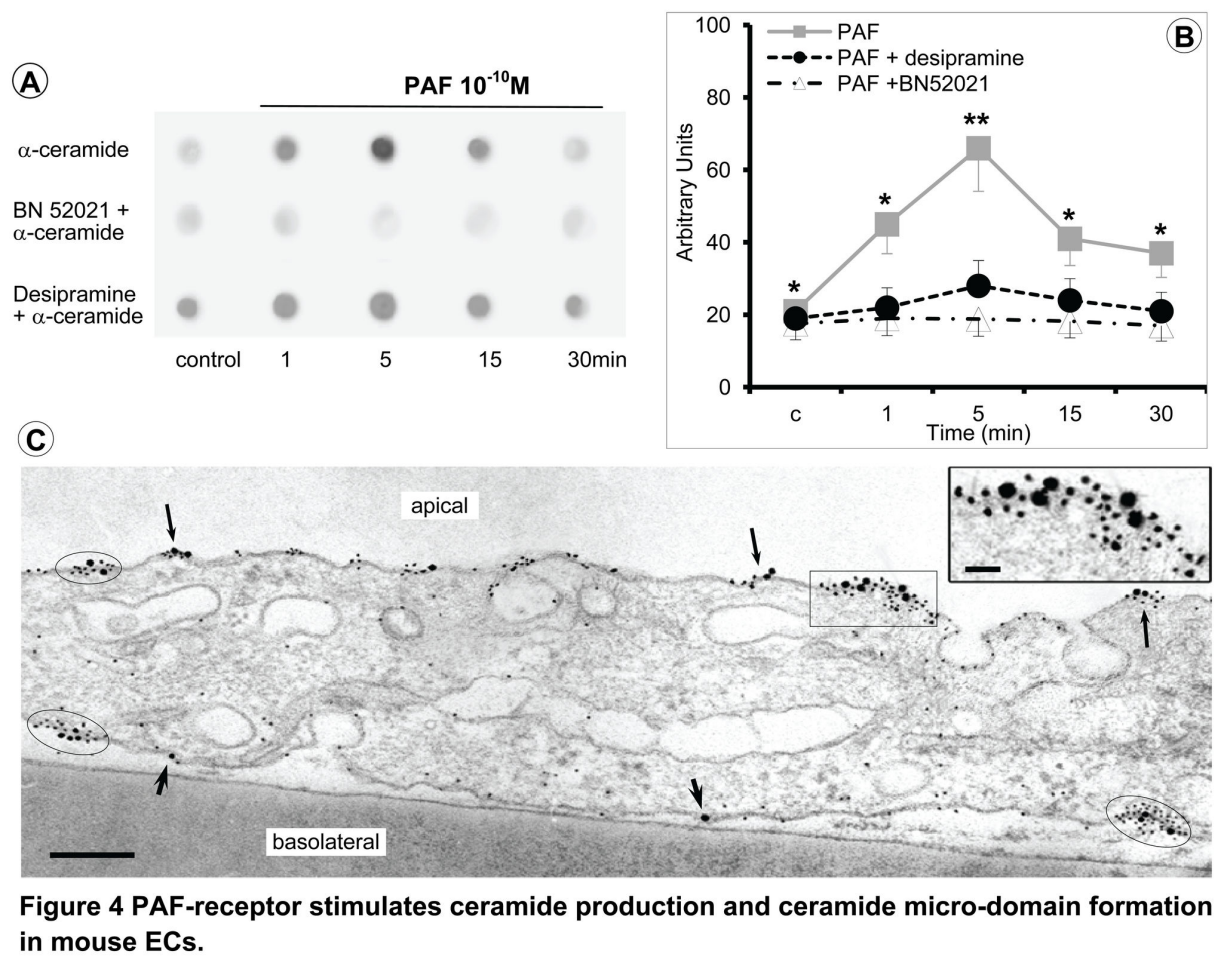
PAF-receptor stimulates ceramide production and ceramide micro-domain formation in mouse ECs. (**A**) PAF treatment of murine ECs induces production of ceramide which is inhibited by pretreatment of ECs monolayers with BN-520521 or with desipramine. (**B**) Densitometric analysis of PAF effects on ceramide production. Results are representative for 4 experiments for each murine PAECs and MVECs cell line and condition. (**C**) Representative TEM micrograph of PAF-treated MVECs showing the immuno-localization of PAF-R (22 nm Au particles) and surface ceramide detected with the anti-ceramide Ab followed by 6 nm Au-tagged secondary Ab; n = 8. Bar = 350 nm. * P < 0.05, and **P < 0.001 when compared with PAF + BN552021.

Since PAF-mediated increase in permeability of mouse ECs monolayers may be mediated by ceramide [14], we next evaluated the ceramide levels in both murine PAECs and MVECs after PAF challenge. Ceramide production in the external leaflet of mouse ECs plasma membrane was determined as in [46]; pilot experiments established that the amount of surface ceramide is proportional with the externally applied bacterial sphingomyelinase (data not shown). Immuno-Dot-Blot with ceramide Ab indicated an increase of ceramide level in the external leaflet of plasma membrane as soon as 1 min, which peaked by 5-15 min, after 10^-10^M PAF, (Figure 4A, upper row). PAF-induced ceramide production was inhibited by pretreating ECs monolayers with PAF-R antagonist BN 52021 (2µM, 45 min, RT), Figure 4A, as well as by treatment with desipramine (10µM, 60 min, RT) an ASM inhibitor, Figure 4A. Quantification of the data using Alpha Innotech AlphaImager software (Figure 4B), indicates a greater than 3-fold increase in the ceramide detected in the external leaflet of the plasma membrane while no significant quantitative alterations were found in the presence of BN 52021 or when ASM was inhibited by desipramine. Altogether these findings demonstrate that PAF-induced ceramide production is dependent on PAF-R and ASM.

Next, we used double immunogold labeling EM -6nm Au particles for ceramide detection and 22nm Au particles PAF-R, to investigate their localization on the mouse ECs plasma membrane. The signal for PAF-R was usually represented by 3-5 PAF-R molecules clustered together (circled areas), and rarely by one Au particle (arrowheads), Figure 4C, c1, while the signal for ceramide was more ample and with a wider distribution. When ECs were exposed to PAF, numerous 6 nm Au particles surrounded PAF-R, suggesting that PAF treatment generates ceramide-enriched micro-domains (0.1-3 µm diameter) around the PAF-R (Figure 4C, c1). The dimensions of ceramide micro-domains were always larger when at least two PAF-R molecules (arrows) subsisted in the same space on the plane of plasma membrane (arrows). In sum, PAF-R displays a clustered distribution on both sides of mouse EC plasma membrane, ceramide has a contained distribution when in the vicinity of the PAF-R in cells exposed to PAF and the signal for ceramide is more widespread that the signal for PAF-R, altogether suggesting that PAF-R engagement results in formation of a ceramide-enriched microdomain centered around the receptor.

### Subcellular distribution of eNOS, PAF-R and Cav1

**Figure 5:**
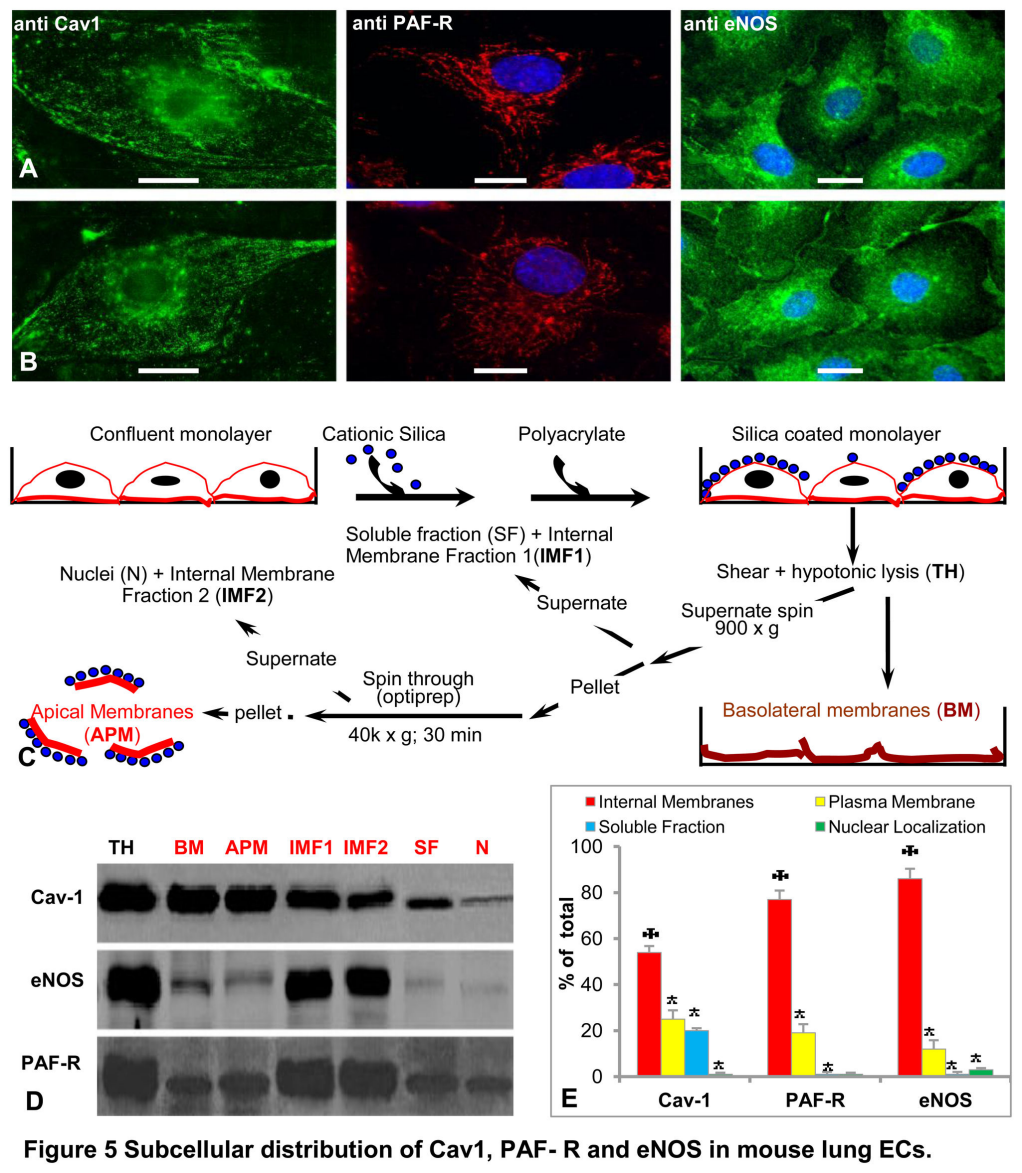
Subcellular distribution of Cav1, PAF-R and eNOS in mouse lung ECs. Representative micrographs of Cav1, PAF-R and eNOS immunolocalization in mouse PAECs (**A**) and MVECs (**B**); Bars: 22 µM. n = 12. (**C**) Overall chart of subcellular fractionation using silica isolation procedure. (**D**) Blots illustrating the distribution of Cav1, PAF-R and eNOS in the sub-cellular fractions isolated as in C. TH = total amount of protein obtained by solubilization of coated monolayers (unfractioned); BM - basolateral plasma membranes, APM - apical plasma membranes; IMF1 - internal membranes that sediment at low gravitational fields as well as mostly of the cytosolic proteins; IMF2 - endoplasmic reticulum and nuclei; n = 8. (**E**) Densitometric analyses of the gels shown in D; * P < 0.05, ✠ P < 0.001.

The distribution of eNOS, PAF-R and Cav1 mouse ECs was assessed first by immunofluorescence in intact PAECs (Figure 5A) and MVECs (Figure 5B), 3 days post-confluence. Cav1/Alexa Fluor 488-conjugated IgG revealed a punctate distribution, slight association with plasma membrane, strong perinuclear signal, most likely in the Golgi area, and a faint signal scattered throughout the cytoplasm, indicating that the bulk of Cav1 resides inside the cells. Immunostaining using PAF-R/Alexa Fluor 594-conjugated IgG indicated the presence of a pool of PAF-R on the plasma membrane, a much larger pool inside the cell as an uninterrupted reticulum and a pool of receptor associated with the nucleus. These findings, along with previously published data [9,11,15] demonstrate that in mouse ECs, the majority of PAF-R localizes at the level of a widespread intracellular network. Finally, the analysis of subcellular distribution of eNOS using eNOS/Alexa Fluor 488-conjugated IgG indicated that in both ECs types the majority of eNOS resides in the perinuclear area, most probably associated with the Golgi; a much weaker signal was found scattered all over the cytoplasm as discrete puncta and a less intense signal was found at the plasma membrane, as previously reported for ECs from heart vasculature [48]. Our data regarding the immuno-localization studies of PAF-R/eNOS inside mouse ECs indicated that the two proteins do not colocalize.

Next, the subcellular distribution of PAF-R, Cav1 and eNOS was studied by silica subcellular fractionation as described in Figure 5C. Protein lysates of the subcellular fractions – apical and basolateral plasma membranes, intracellular membranes, nuclear fraction as well as the soluble fraction were subjected to electrophoretic separation and Western blot analyses followed by densitometry. Cav1 is present in both, apical and basolateral endothelial plasma membrane fractions as well as in the internal membranes. Cav1 is present also as a soluble fraction and the nuclear fraction of murine ECs (Figure 5 D). Quantitative analysis demonstrates that the majority of Cav1, 54%, is associated with the internal membranes, 25% is found at the level of plasma membrane, about 20% in the soluble fraction, whereas <1% in the nuclear fraction (Figure 5 E). These data demonstrate that in mouse ECs most of Cav1 is associated with different cellular membranes and only 20% resides inside as a soluble fraction, confirming previous studies in different types of ECs [49]. The eNOS distribution, Figure 5 D, demonstrates that the bulk of eNOS associates with the internal membranes of the cell, a small fraction is found at the level of both apical and basolateral plasma membranes, while only minute amounts were found as soluble fraction and associated with the nuclear fraction. Quantitative analysis of eNOS distribution, (Figure 5 E), indicated that 86% is associated with the internal membranes, only 11.8% is associated with the plasma membrane, 1% seems to exist as soluble fraction, while less than 1% is associated with the nuclear fraction. Our data substantiate the fact that under unstimulated conditions, there is at least four times more eNOS enzyme inside the cells than at the plasma membrane. Quantitation of PAF-R association with different subcellular compartments (Figure 5D, E), indicate that 77% of receptor resides inside the cell associated with the intracellular membranes, 19% is associated with the apical and basolateral plasma membranes, 3% is associated with the nucleus and a tiny amount (<1%) is found in the soluble fraction. As the distribution of PAF-R was never before studied in mouse ECs, we concluded that its distribution is similar with what was described in other types of ECs [9,15].

We next addressed, in similar studies, the quantitative alterations in the subcellular distribution, of the three proteins, after PAF (10^-10^M) challenge. We found that: i) there are no statistical changes in the distribution of Cav1, ii) a small redistribution of eNOS (84% in intracellular membranes, 13.5% in the plasma membrane and 1.5% in soluble fraction) occurred, but without statistical significance, and iii) the PAF-R redistribution (53% inside the cells, 38% in apical and basso-lateral membranes, and 5% in nuclear fraction) reached statistical significance (P< 0.005). Based on these data we concluded that the overall subcellular distribution of the three proteins in mouse ECs follows the same pattern described for ECs isolated from different species.

### Sub-domains association of PAF-R, eNOS and Cav1 on the lateral plan of plasma membrane

**Figure 6:**
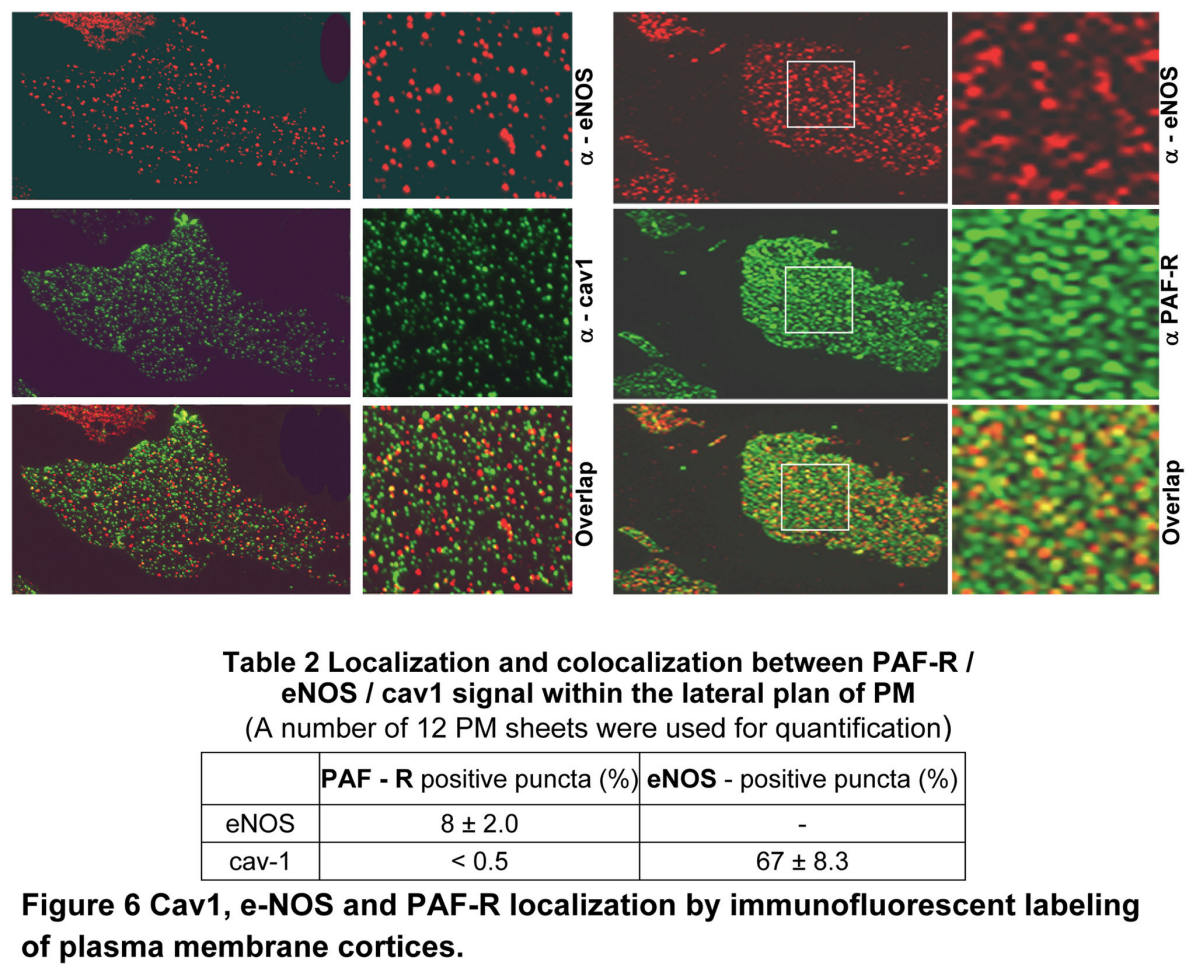
Cav1, e-NOS and PAF-R localization by immunofluorescent labeling of plasma membrane cortices. Representative images illustrating the localization of eNOS/PAF-R (**A**) and eNOS/Cav1 (**B**) as revealed by immunostaining of plasma membrane cortices prepared from MVECs, immunostained with primary antibodies followed by appropriate reporters conjugated to either Alexa Fluor 488 or Alexa Fluor 594. Bar = 22µM (a1 - a3; b1 - b3); 35µM (insets); n = 15.

**Figure 7:**
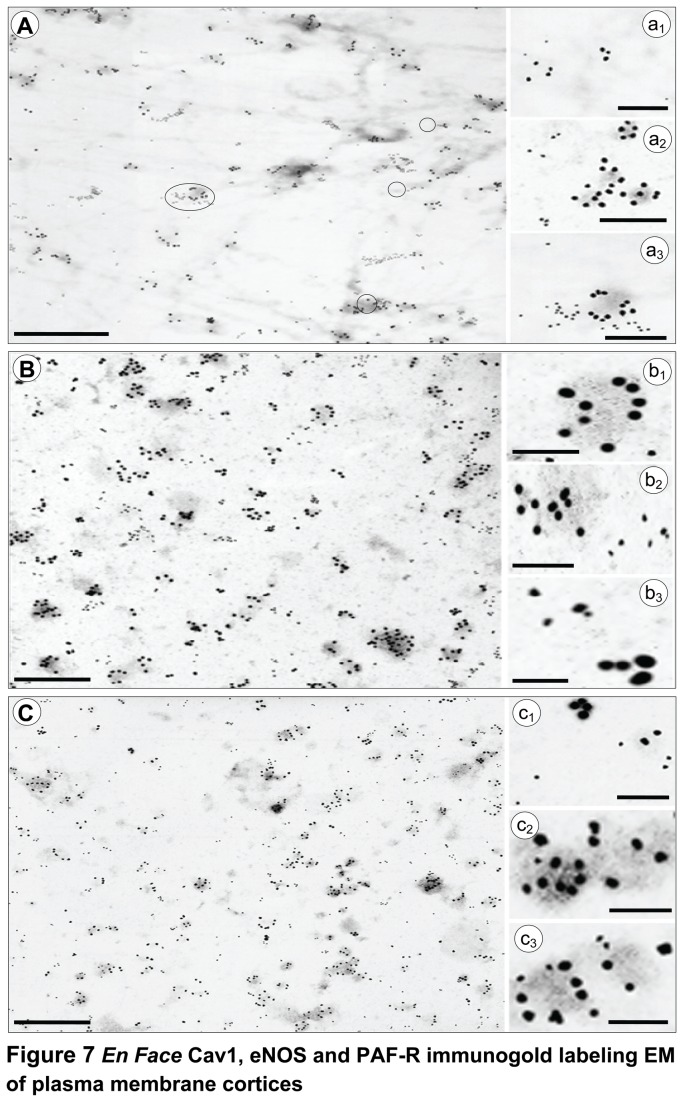
“*En face*” Cav1, eNOS and PAF-R immunogold labeling EM of plasma membrane cortices. (**A**) The overall distribution of 5 nm Au PAF-R and 15 nm Au eNOS on the plane of plasma membrane. The majority of PAF-R resides outside recognizable vesicular profiles (a1) independent of eNOS, while the signal for eNOS is associated with vesicular profiles (a2), and seldom co-localized with PAF-R (circles in the main panel, out of which one is magnified in a3); n = 4. (**B**) 5nm Au PAF-R and 15 nm Au Cav1 labeling confirm that there is no colocalization between the two proteins. Whereas Cav1 display its characteristic “ring shape” distribution over the caveolae profiles (b1), the signal for PAF-R is never close to the caveolae (b2), and the two proteins exists as small clusters (> 2gold particles outside vesicular profiles (b3); n = 6. (**C**) 5 nm Au eNOS and 15 nm Cav1 labeling of plasma membrane patches documents the existence of the two proteins residing outside of any identifiable vesicular profile (c1), the presence of vesicular profiles labeled only by Cav1 (c2), and their co-localization at the level of several vesicular profiles (c3). n = 5. Bars: 100 nm (A, B, C) and = 40 nm (a1 – a3; b1 –b3; c1 –c3).

Usually, a cell surface receptor is preferentially distributed in defined micro-domains of the plasma membrane; it is assumed that the residence domain influences and even determines the outcome of receptor’s intracellular signaling. Since existing data point out to the existence of a functional partnership PAF-R / eNOS, two proteins distributed in different subcellular domains, and since it is rationalized that eNOS has exclusive caveolar localization, we sought to determine the spatial relationship between PAF-R, eNOS and Cav1, when on the plasma membrane, using fluorescence microscopy on plasma membrane patches. Basolateral membrane patches were prepared and immunostained as in [40]. This “en face” immunostaining approach, applied on unstimulated ECs, revealed a similar punctate distribution for eNOS (Figure 6A, a1) and PAF-R (Figure 6A, a2) and made obvious their partial co-localization (Figure 6A, a3). Highly magnified images (boxed area, a2) pointed out to a more elaborate spatial distribution of PAF-R in the plane of plasma membrane, that is coexistence of individual puncta with small aggregates of more than 2 puncta, that have the tendency to coalesce; most probably this pattern represents a sum of the signal generated by several receptor molecules clustered together. The quantitative analyses of PAF-R / eNOS distribution indicated only 8% colocalization (Table 2), while the majority of the signal (91%) for both eNOS and PAF-R resides in separate micro-domains of plasma membrane proper. The “en face” immunostaining used revealed a punctate distribution for both eNOS (Figure 6B, b1) and Cav1 (Figure 6B, b2), significantly stronger for Cav1 and as expected revealed their co-localization in the plane of plasma membrane (Figure 6B, b3). Moreover, eNOS displays different sized puncta populations - many tiny puncta that may represent individual eNOS molecules and fewer larger puncta, which may be generated by more amassed molecules - scattered all over plasma membrane surface. Cav1-positive puncta displayed a higher density (b2, boxed area). The merged image (Figure 6B, b3) shows however that some Cav1-positive puncta do not bear the signal for eNOS and some eNOS-positive puncta do not co-localize with Cav1. Quantitative analysis of their distribution indicated: i) 60% Cav1/eNOS co-localization (Table 2), ii) 2.5-fold more signal for Cav1 than for eNOS, iii) existence of an eNOS fraction (>9%), not associated with Cav1, suggesting its localization outside of the caveolae micro-domains. Similar studies performed for Cav1/PAF-R distribution on plasma membrane patches indicated that less than 0.5% of the Cav1-positive puncta colocalized with PAF-R, a value with no statistical significance most probably reflecting a bleed through fluorescence from one channel to the other (not shown). The distribution and the movement of Cav1 and eNOS in and out of lateral plane of plasma membrane patches didn’t change significantly, after PAF stimulation. On PAF stimulated patches, the movement of Cav1 and eNOS didn’t reached statistical significance, while the redistribution of PAF-R was the only event with statistical significance, and we always found an increase (> 28%) in the number of PAF-R. Despite its power, this approach cannot provide answers to two fundamental questions: i) whether eNOS and Cav1 localize outside recognizable vesicular domains in the lateral plane of plasma membrane, and whether there is any co-localization among Cav1, eNOS and PAF-R outside identifiable vesicular carriers. To address these concerns a detailed study of their distribution was performed by double-EM immunogold staining of plasma membrane patches prepared from PAF-exposed ECs grown on poly-l-lysine-coated EM gold grids. Immunostaining was performed as follows: PAF-R rabbit-pAb/eNOS mouse-mAb, Figure 7A, PAF-R rabbit-pAb/Cav1 mouse-mAb, Figure 7B, and anti-eNOS rabbit-pAb/Cav1 mouse-mAb, Figure 7C, followed in each case by appropriate reporters, 5nm Au-conjugated anti-rabbit IgG and 15 nm Au-conjugated anti-mouse IgG and standard EM staining. Under these conditions, we considered that two proteins co-localize if the distance between the two size Au particles was 20nm or less. Similar data were obtained on patches prepared from unstimulated mouse ECs. Co-distribution analyses demonstrated that PAF-R is preferentially localized on the plasma membrane proper (> 99%) not associated with any recognizable vesicular profile (Figure 7A, a1); Au particles constantly located at a distance of more than 30 nm of any vesicular profile (Figure 7a2). A hefty eNOS signal (~85%) associated with vesicular profiles (Figure 7A, a2, a3). Only 8% of the signal for both proteins on the plasma membrane, not associated with vesicular profiles, existed at a distance of < 20 nm (Figure 7A, a3, circles) indicative that PAF-R/eNOS co-localize only outside of recognizable vesicular profiles. When 5 nm Au-PAF-R and 15 nm Au-Cav1 distribution was assessed (Figure 7B), we found: i) no co-localization between the two proteins (>99% of the Au particles was found at a distance of more than 20 nm), ii) Cav1 staining revealed the characteristic ring-shape distribution over the vesicular profiles (Figure 7, b1), iii) the signal for PAF-R always was more than 20 nm in regard to the vesicular profiles labeled by Cav1 (Figure 7, b2) and iv) PAF-R exists on the plasma membrane as small patches formed by two or more molecules (Figure 7B, b3) and occasionally as solitary particles or as pairs scattered all over plasma membrane (Figure 7B). The distribution of 5 nm Au-eNOS and 15 nm Au-Cav1 on the lateral plane of plasma membrane (Figure 7C), revealed that: i) significantly, there is signal for both proteins outside of vesicular profiles (Figure 7C, c1), ii) there are vesicular profiles labeled only by Cav1 (Figure 7, c2) and iii) the two proteins co-localize at the level of caveolar profiles (Figure 7, c3); Note the heavy “stripe-like” Cav1 labeling and the less abundant eNOS labeling. Quantitative analyses indicated that: i) 60% of all eNOS co-localizes with Cav1, ii) 51% of all Cav1 co-localizes with eNOS, and iii) their co-localization represents less than 22% of the total number of Au particles counted.

**Table 2:** Co-localization of PAF-R/eNOS/Cav1 within the lateral plan of plasma membrane.

	**PAF-R** positive puncta (%)	**eNOS** - positive puncta (%)
eNOS	8 ± 2.0	-
cav-1	< 0.5	60± 8.3

Twelve plasma membrane sheets were used for quantification.

## Discussion

The activation of ECs by “classic” transmembrane” receptor ligands such as bradykinin, acetylcholine and PAF is accompanied by the elevation of intracellular calcium and subsequent release of NO [29,31,50]. PAF is recognized as one of the most potent vasoactive [9] and inflammatory [51] mediators. However, the complete registry of PAF-induced morphological alterations of endothelial barrier as well as the molecular pathways triggered by PAF-R engagement have not been fully described [8–10,12,52].

The reduced synthesis or bioavailability of NO which is invariably linked to compromised blood vessel function is a hallmark of cardiovascular disease [53]. The amount of NO produced by eNOS is tightly controlled by the interplay of several distinct posttranslational modifications, including phosphorylation, protein-protein interactions, and subcellular localization [25,29]. These controls have evolved to facilitate the delivery of sufficient quantities of NO at the right place and time to initiate the appropriate signaling response.

Using isolated mouse lung ECs we have established a direct correlation between PAF treatment and the production of NO by ECs. Post-confluent monolayers of MVECs treated with 10^-10^M PAF and loaded with DAF-2DA showed a time-dependent production of NO, which peaked between 5-15 min, started to return toward basal levels by 30 min and reached basal levels by 60 min; similar results were obtained with mouse PAECs. In both types of mouse ECs, NO production was blocked by L-NAME pretreatment, consistent with the idea that PAF-induced NO production is eNOS-dependent. These results are in contrast with previous reports showing that PAF treatment (perfusion) decreases NO production in the lung vasculature [54]. The methodologies used (whole organ perfusion versus isolated ECs), the species in which NO production was examined (rat versus mice) and the lack of NO production measurements could perhaps explain the contradictory results. A real-time recoding of PAF-induced NO production in both types of mouse ECs using the porphyrinic electrode, that apparently overestimates NO concentration [55], allowed us not only to show that these cells respond with a burst of NO, but also to infer about possible kinases and other enzymatic systems that modulate its production. The results obtained with a wide range of inhibitors show that PAF-induced NO is shaped by PKC, AMPK, PKA, Akt, cSrc kinases known to post-translationally modify eNOS and control NO production in other cell types [24–26]. However, detailed mechanisms by which different kinases shape the cellular outcome of NO production were beyond the purpose of this work.

In addition, PAF-induced NO production is abolished by BN 52021, fact that demonstrates it dependence on the PAF-R. To substantiate these findings, a detailed analysis of PAF-induced phosphorylation of eNOS was performed using available phospho-specific Abs for different phosphorylation sites of the enzymes. We provide data demonstrating a robust and sustained phosphorylation/dephosphorylation of Ser^1177^, Ser^615^, Ser^633^ and Thr^495^ paralleled by an increase/decrease in NO production. Moreover, immunoprecipitation of eNOS with PY-20 Abs along with its dependence on cSrc and PAF-R, also demonstrate that PAF-induced Tyr phosphorylation is part of enzyme post-translational modifications involved in shaping NO output. Thus, our data substantiate PAF capability to induce NO production as a consequence of eNOS activation [18,29,32].

While eNOS must resides in caveolae to be a functional molecule [23,29,30,56–58], PAF-R is exclusively localized on plasma membrane. No spatial relationship between the two molecules was demonstrated, so far. Thus, now we demonstrate how two molecules, residing in separate plasma membrane micro-domains can interact and generate NO. We showed that: i) PAF-R engagement generates ceramide species on the outside leaflet of plasma membrane, ii) the ceramides are organized in the lateral plane of plasma membrane as distinctive micro-domains, iii) ceramides production and ceramide micro-domain formation are PAF- and to a larger extent, ASM-dependent, and iv) ASM inhibition hampered NO production. Moreover, PAF-induced accumulation of ceramides on the plasmalemma proper was responsive to PAF-R inhibitor BN 52021 and to desipramine, thus connecting NO production to ceramides formation. Corroborating PAF-induced ceramides production in the outer leaflet of mouse ECs plasma membrane with the established role of ASM-generated ceramide at the cell surface in the reorganization of membrane domains [59,60], we advance the idea that activation of ASM following PAF-R engagement triggers production of ceramide molecules around the receptor, which by interacting with each other exclude other lipid species and create a PAF-induced ceramide micro-domain.

Based on the inhibition of NO production by ASM inhibitor desipramine and as demonstrated for other ceramide-signaling spots initiated by G-protein-coupled receptors [60], the PAF-induced ceramide micro-domains function as signaling platform for PAF-R. When considered in conjunction with the exclusive localization of PAF-R on the plasma membrane, PAF-induced assembly of ceramide micro-domains provides the necessary structural base to explain how the receptor signals when residing at the level of a membranous structure. However, it remains to be established whether and if: i) the newly generated ceramide molecules influence the PAF-R when embedded in the new micro-domain, ii) their enhancing and/or lowering effect on the threshold of receptor signaling, iii) they participate in receptor internalization, iv) the same ceramide rafts are responsible for PAF signaling when the receptor is localized in the intra-cellular tubulo-vesicular system and on the nuclear membrane, and v) the ASM is the only ceramide-generating enzyme involved. PAF-induced ceramide signaling platforms in the plasma membrane could also explain how a receptor which is not associated within any known vesicular carriers can signal and be internalized.

The addition of ASM to the enzymatic systems activated by PAF is a great contribution to the molecular mechanisms by which PAF exerts its actions; however, the mechanism(s) by which ceramides participate in PAF-increased vascular permeability should be further investigated.

Although the spatio-temporal distribution of Cav1, PAF-R and eNOS in cultured mouse ECs studied by fluorescence microscopy gave an overview of their cellular location, the dimension of their quantitative allocation among different subcellular spaces was still missing. Consequently, our detailed quantitative biochemical analyses of the subcellular distribution of eNOS and PAF-R on subcellular fractions obtained after silica coating, clearly demonstrated that most of eNOS (70%) and of PAF-R (85%) are found inside the cells. While our data confirm previous reports showing that majority of eNOS associates with the internal cellular membranes [48], they didn’t frankly addresses the relationship between eNOS/PA F-R within the cell; yet we didn’t find colocalization inside ECs between the two proteins.

The fact that most of the studies published so far made the assumption that eNOS is associated with plasma membrane in order to generate the NO concentration necessary for functional efficacy [56,61,62] and our report demonstrating a different subcellular distribution of eNOS and no co-localization with PAF-R inside the cell, shouldn’t be seen as a contradiction, as long as in the plane of plasma membrane the two proteins showed co-localization. The 8% overlapping between eNOS and PAF-R signals on the membranes of mouse ECs outside any recognizable vesicular profiles corroborated with the localized PAF-induced production of ceramide, suggest that newly induced micro-domains are the signaling platforms used by the receptor for signaling and internalization. PAF-R/eNOS co-localization at the level of ceramide based domains adds a new facet to the fields of PAF-R and eNOS and also may explain unanswered questions about PAF-R signaling, such as eNOS activation, its internalization and trafficking.

Immunostaining of plasma membrane cortices, at light and EM level for Cav1 and eNOS showed that there is a >60% overlapping of their signals; however, a small signal for Cav1 was found outside identifiable vesicular carriers, and noticeably, a large amount of eNOS signal was not associated with the caveolar profiles. These findings ascertain the existence of eNOS signal outside of a caveolar fraction [63], but challenge the current view of exclusive Cav1 and eNOS distribution on the plasma membrane only in caveolae [62,64]. Our data are in agreement with immuno-EM studies showing the existence of eNOS signal in plasmalemma micro-domains that have a few or are devoid of caveolae [63,65] and with studies showing its localization at the leading edge of migrating cells from where the caveolae are excluded [66]. By corroborating our findings with already published work we demonstrate that a hefty fraction of eNOS exists in the plasma membrane outsides caveolae; yet several issues still remain: i) how this fraction is involved in NO production, ii) how is its enzymatic activity regulated, and iii) whether the mechanism(s) responsible for the residence outside caveolae are similar with the ones responsible for its existence only in caveolae.

Published data show that caveolae may facilitate some PAF-R signaling events by placing the receptor in close proximity of Cav1 in living cells [67]. However, by blurring the distinction between caveolae and Cav1, by not providing a dimension for “their close proximity” and by speculating on PAF effects in cells lacking caveolae, we consider that such conclusion lacks the most important piece of evidence - the ultrastructural localization between the two proteins. It has been reported that PAF induces via ASM an increase in Cav1 and eNOS amounts in the so called “caveolar fraction”; however, no increase in the number of caveolae or changes in their subcellular distribution and size were reported [68]. Even though, while no conciliatory explanation between the biochemical findings (increase in the amounts) and the morphological data (no modification of caveolae number) were provided, the final conclusion of that work was that the abundance of Cav1 and eNOS was increased by PAF treatment. As studied by us, using silica coating of isolated mouse ECs, there is no increase in Cav1 and eNOS levels in cells treated with PAF, and thus the simple explanation of such contradictory findings is “the guilty by insolubility” comportment of different proteins when extracted in the presence of detergents, according to which “detergent insolubility and density gradient centrifugation of such cell lysates, are not methods to establish caveolar localization of proteins and lipids” [69].

The use of plasma membrane cortices is sensitive enough to provide quantitative information about the movement of Cav1 and eNOS after PAF treatment; our data show only a limited movement for eNOS, no redistribution of Cav1, and enrichment in PAF-R. All previous studies have used whole cell staining or classical subcellular fractionation to prove that eNOS and Cav1 are relocated and in some cases quantification of their relocation was attempted. While the fluorescence microscopy lacks the necessary resolution for a precise assignment of a protein to a subcellular compartment and as long as no super-resolution fluorescence microscopy was used for their detection, these results suffer from the same flaw, lack of definitive proof of their movement from one subcellular compartment to another. Thus, our data obtained by EM immunostaining of plasma membrane cortices obtained from ECs exposed to PAF, revealed that the number of Cav1-positive puncta/µm^2^ of plasma membrane patches is not changed; in other words we do not have more caveolae per unit surface of ECs. This is also true for experiments made *in vivo* when the number of luminaly, apparently free in cytoplasm or abluminaly open caveolae per unit of endothelial volume is not changed after PAF. Thus, the set of data obtained on plasma membrane cortices and related to the number of caveolae that do not change after PAF, is in agreement with similar published morphologic data obtained by topical application of PAF [9], and with data obtained by perfusing the lung vascular bed with PAF [10]. However, for eNOS relocation our data show a loss of < 4% of the signal after PAF, but this value did not reached statistical significance. Hence, better designed and more dynamically oriented studies are necessary to address these issues.

In sum, the existence on the plasma membrane of Cav1 and eNOS residing outside recognizable vesicular profiles in the lateral plane of plasma membrane, the colocalization between PAF-R and eNOS and the localized ceramides production after PAF-R engagement leading to the formation of micro-domains on the plasmalemma proper, are new findings which allow to explain how it’s possible for two proteins not residing in identifiable vesicular carriers, to interact and to trigger signaling events.
